# Editorial: Unlocking the nexus of bioactive components, nutrition, and nutrigenomics in age-related disorders

**DOI:** 10.3389/fnut.2025.1597658

**Published:** 2025-04-25

**Authors:** Antoni Sureda, Xavier Capó

**Affiliations:** ^1^Research Group on Community Nutrition and Oxidative Stress, University of the Balearic Islands-IUNICS, Palma de Mallorca, Spain; ^2^Health Research Institute of Balearic Islands (IdISBa), Palma de Mallorca, Spain; ^3^CIBEROBN (Physiopathology of Obesity and Nutrition), Instituto de Salud Carlos III, Madrid, Spain

**Keywords:** nutrigenomics, bioactive compounds, healthy aging, age-related disorders, personalized nutrition, dietary interventions

Aging is a natural and complex biological process that involves a multitude of physiological, molecular, cognitive and social changes. As individuals age, their susceptibility to a range of chronic diseases, such as cardiovascular disease, metabolic dysfunction, neurodegenerative conditions, and musculoskeletal decline, increases significantly (1). These age-related diseases not only impact quality of life but also pose substantial challenges to global healthcare systems, making the search for effective interventions more important than ever. In this context, nutrition and bioactive compounds have emerged as promising avenues for mitigating the adverse effects of aging, offering potential strategies to extend health span and delay the onset of debilitating conditions.

The link between nutrition and aging is well established, with growing evidence that specific nutrients and dietary patterns can modulate biological pathways and gene expression involved in aging. Bioactive compounds have demonstrated their ability to influence inflammation, oxidative stress, and mitochondrial function to support metabolic health and cognitive function (2, 3). These compounds are increasingly being recognized for their role in promoting health during aging. Furthermore, nutrigenomics is providing valuable insights that may lead to personalized strategies for healthy aging and the prevention of age-related diseases (4, 5).

This Research Topic brings together a collection of studies that explore how various bioactive components of the diet impact aging-related processes and chronic conditions. The research presented in this volume provides compelling evidence on how various bioactive compounds can modulate aging-related processes and prevent or delay the onset of chronic conditions associated with aging.

In this Research Topic, eight distinct studies are presented, each contributing to the relationship between diet, bioactive compounds, and aging.

Several studies in this Research Topic examined the critical role of vitamins in muscle function and musculoskeletal health, highlighting their potential to prevent age-related decline. One study (Qi et al.) investigated the impact of vitamin D supplementation on muscle strength in middle-aged and elderly individuals, demonstrating that adequate vitamin D intake, combined with lifestyle interventions, enhances lower limb and grip strength, thereby reducing the risk of falls and frailty. Notably, participants who received vitamin D supplementation along with health education showed significant improvements in serum calcium levels and muscle strength, reinforcing the essential role of vitamin D in maintaining musculoskeletal health in the aging population. Another study (Yang et al.) examined the association between vitamin B intake and muscle mass loss in younger individuals, particularly in relation to early-onset sarcopenia. The findings suggest that higher intakes of B vitamins, specifically B1 (thiamine) in men and B2 (riboflavin) in women correlate with a lower risk of sarcopenia. This underscores the importance of early dietary intervention to preserve muscle integrity and prevent premature muscle loss across different age groups.

Following these insights into vitamin-related muscle health, another study explored the neuroprotective effects of dietary vitamin K (Luo and Lin). The researchers found a significant negative association between vitamin K intake and serum neurofilament-light chain (NfL) levels, a biomarker of neurodegenerative disease. Individuals with higher vitamin K intake exhibited lower NfL levels, suggesting a potential role for this vitamin in cognitive health and the prevention of neurodegenerative disease, particularly in middle-aged and older adults.

In addition to musculoskeletal and neurological health, this Researh Topic also addressed the impact of diet on inflammation and age-related diseases. One study (Zhang et al.) investigated the association between the dietary inflammatory index (DII) and the risk of age-related cataracts, revealing that a pro-inflammatory diet is significantly linked to increased cataract prevalence. Using NHANES data, the authors found that individuals with higher DII scores—indicating a diet rich in inflammation-promoting foods—were more likely to undergo cataract surgery. These findings emphasize the importance of anti-inflammatory dietary patterns, including those rich in omega-3 fatty acids, polyphenols, and fiber, in the prevention of age-related ocular disease.

Similarly, another study (Pei et al.) examined the role of nutrition and inflammation in elderly patients with brain abscesses. Their findings highlight that nearly half of the assessed patients were at risk for malnutrition, a condition closely linked to elevated inflammatory markers, such as C-reactive protein. Malnutrition was associated with increased comorbidities and poorer recovery outcomes, with lower Geriatric Nutritional Risk Index (GNRI) values correlating with poorer clinical recovery. These results suggest that nutritional assessment and intervention should be a key component in the management of neurological infections in the elderly. Expanding on the relationship between diet and inflammation, the association between dietary magnesium intake and pelvic inflammatory disease (PID) has been investigated (Chen et al.). Their study found a significant inverse relationship between dietary magnesium intake and PID risk, particularly in older women. Higher magnesium intake was associated with a lower likelihood of PID, highlighting the potential role of magnesium in modulating inflammation and immune function in gynecological health. These findings reinforce the broader significance of dietary factors in the management of inflammation-related conditions across different organ systems.

Further extending the discussion on diet and inflammation, a Mendelian Randomization study (Luo et al.) examined the causal relationship between macronutrient, antioxidant, mineral, and vitamin intake with childhood asthma (CA). Interestingly, the study found that higher sugar intake is inversely correlated with the risk of CA, while higher fat consumption, magnesium intake, and serum vitamin D levels are positively associated with a higher risk of CA. Although this research focused on pediatric populations, its findings contribute to a broader understanding of how dietary components influence inflammatory pathways, which may also have implications for age-related conditions.

Finally, a study assessing the plant-based dietary index (PDI) and cardiovascular disease (CVD) risk in elderly Chinese individuals (Hou et al.) revealed a significant inverse association between higher adherence to a plant-based diet and reduced CVD risk. Notably, these benefits appear to be independent of other lifestyle factors such as BMI, smoking, alcohol use, or exercise. These findings reinforce the importance of plant-based dietary patterns in promoting cardiovascular health in aging populations and provide strong support for dietary recommendations that emphasize increased consumption of plant-derived foods.

In summary, although much work remains to be done, the studies presented in this Researh Topic provide robust evidence for the pivotal role of nutrition and bioactive compounds in mitigating age-related diseases. These findings highlight how specific nutrients and personalized dietary interventions can contribute to promoting healthy aging and preventing chronic diseases. Moving forward, continued exploration of dietary strategies and their interactions with individual genetic and biological factors will be essential to refine therapeutic approaches and design targeted interventions for aging populations worldwide.
